# Effects of comprehensive group music therapy on affect and social functioning in patients with schizophrenia undergoing community-based rehabilitation: a preliminary study

**DOI:** 10.3389/fnhum.2025.1645981

**Published:** 2025-11-26

**Authors:** Peikun Hong, Chao Xue, Junping Lu, Mengying Wang, Hongrun Pan, Hua Shao

**Affiliations:** 1 Department of Psychiatry, The Third Hospital of Jinjiang, Quanzhou, Fujian, China; 2 School of Educational Science, Quanzhou Normal University, Quanzhou, Fujian, China; 3 School of Teachers College, Shihezi University, Shihezi, China

**Keywords:** community rehabilitation, schizophrenia, comprehensive group music therapy, affect, social functioning

## Abstract

**Background and objective:**

Most individuals with schizophrenia reside in the community, where they frequently encounter difficulties related to emotional problems and social functioning– critical areas of concern in the rehabilitation process. This study aims to investigate the effects of a comprehensive group music therapy intervention on the emotional state and social functioning of individuals with schizophrenia undergoing community rehabilitation.

**Methods:**

A total of 28 individuals with schizophrenia in community rehabilitation were randomly assigned to either the music intervention group (*n* = 14) or the control group (*n* = 14). The music intervention group participated in an 8-week comprehensive group music therapy intervention, while the control group continued with routine family life. The music therapy program was culturally adapted to the local community context, incorporating familiar regional music and dialect. The Positive and Negative Affect Schedule (PANAS) served as the primary outcome to assess affective changes, and the Social Disability Screening Schedule (SDSS) served as the secondary outcome to evaluate social functioning. Both measures were administered pre- and post-intervention. In addition, some participants in the intervention group completed semi-structured interviews to explore changes in social functioning and affect.

**Results:**

Following the intervention, the music therapy group showed significant improvements in positive affect, social activities and self-care, as measured by the PANAS and SDSS, respectively. In contrast, no significant pre-post changes were observed in the control group. Semi-structured interviews further supported these findings, with participants in the intervention group reporting enhanced emotional state and improved social functioning.

**Conclusion:**

Comprehensive group music therapy appears to be an effective intervention for improving emotional states and social functioning in individuals with schizophrenia engaged in community rehabilitation. This culturally adapted intervention model demonstrates its potential for broader application.

## 1 Introduction

Schizophrenia is a severe mental disorder characterized by profound impairments in cognition, emotion, and thought processes, often accompanied by significant deficits in social functioning (Min et al., 2015). According to the World Health Organization, schizophrenia affects approximately 24 million individuals worldwide, with nearly 80% undergoing rehabilitation in community settings (WHO, 2024). This poses considerable challenges for community-based psychiatric rehabilitation. Pharmacological treatment remains the primary approach in such settings; however, these treatments are frequently associated with side effects and demonstrate limited efficacy in restoring social functioning and enhancing emotional well-being (Leucht et al., 2024; Jiang et al., 2025). As a result, there is growing interest in adjunctive rehabilitation strategies, among which music therapy has emerged as a promising non-pharmacological intervention.

Music therapy is a psychosocial intervention wherein trained therapists use diverse musical experiences and therapeutic relationships to facilitate patient recovery (Shoemark et al., 2015). It has been widely applied in the rehabilitation of individuals with schizophrenia (de Witte et al., 2022). Evidence from prior studies suggests that both individual and group music therapy can alleviate multiple symptoms and improve functioning in individuals with schizophrenia. Specifically, music therapy has been shown to reduce psychiatric symptoms (Ahmadvand et al., 2024; Jia et al., 2020; Kosugi et al., 2019; Lam et al., 2023; Pedersen et al., 2021; Peng et al., 2010; Silverman, 2003; Talwar et al., 2006; Tseng et al., 2016; Yang et al., 1998), enhance cognitive function (Ertekin Pinar and Tel, 2019; Kosugi et al., 2019), alleviate depression (Gassner et al., 2022; Jia et al., 2020; Lassner et al., 2024; Lu et al., 2013), improve emotional behavior (Lee and Lee, 2020), promote social functioning (Gassner et al., 2022; Geretsegger et al., 2017; Lam et al., 2023; Lee and Lee, 2020), enhance quality of life (Geretsegger et al., 2017; Jia et al., 2020; Lam et al., 2023; Lassner et al., 2024), and mitigate sleep disturbances (Lu et al., 2022). Moreover, neurophysiological studies have documented music therapy-induced changes in brain function, including alterations in attention-related P300 event-related potentials (Ahuja et al., 2020), modulation of resting-state EEG patterns (Wang et al., 2024), and improvements in gray matter volume and default mode network connectivity (Ivanova et al., 2022; Yao et al., 2020). These interventions often include a range of activities such as passive listening, singing, instrument playing, and songwriting, with group formats being particularly prevalent given the high prevalence of social withdrawal among individuals with schizophrenia (Wang et al., 2024). By enabling patients to autonomously select preferred music and engage in cooperative music-making within the group, group music therapy promotes autonomy, fosters a sense of belonging, enhances perceived social support, and encourages meaningful social interaction (Geretsegger et al., 2017).

Despite these promising findings, most existing studies have been conducted in inpatient settings, with relatively limited exploration of group music therapy within community-based rehabilitation programs. Moreover, prior research has predominantly focused on symptom reduction—such as changes in Positive and Negative Syndrome Scale (PANSS) scores (Pedersen et al., 2021; Wang et al., 2024)—while giving comparatively less attention to improvements in emotion and social functioning. In inpatient settings, music therapy typically targets acute symptom management (Gold et al., 2009), whereas in community rehabilitation, the emphasis shifts toward the restoration of social functioning and reintegration into the community (Geretsegger et al., 2017). Accordingly, group music therapy in community contexts may benefit from larger group formats and intervention designs that foster interpersonal interaction. Furthermore, unlike the high heterogeneity often observed among inpatients, individuals in community rehabilitation tend to share similar socio-cultural backgrounds, underscoring the importance of incorporating local cultural elements into music therapy programs.

In addition to these contextual considerations, most previous studies have relied on a single modality of music therapy (e.g., active or passive format). Few have systematically examined the integration of multiple approaches to test for potential additive or synergistic effects (e.g., Tang et al., 2020; Zhang et al., 2022). Developing such comprehensive group-based music therapy programs may be particularly suitable for community-dwelling individuals with schizophrenia, whose needs extend beyond symptom reduction to include social connectedness and emotional adjustment. Similar perspectives have also been highlighted in music therapy research with other populations (Kayaaslan and Lök, 2025; Ting et al., 2024).

To address these gaps, the present study developed a culturally adapted, comprehensive group music therapy program grounded in Wheeler’s theoretical framework (Unkefer and Thaut, 1990; Wheeler, 1983), tailored to the clinical and socio-cultural characteristics of individuals undergoing community-based schizophrenia rehabilitation. The aim was to examine the effects of this intervention on participants’ emotional state and social functioning.

## 2 Materials and methods

### 2.1 Participants

**FIGURE 1:**
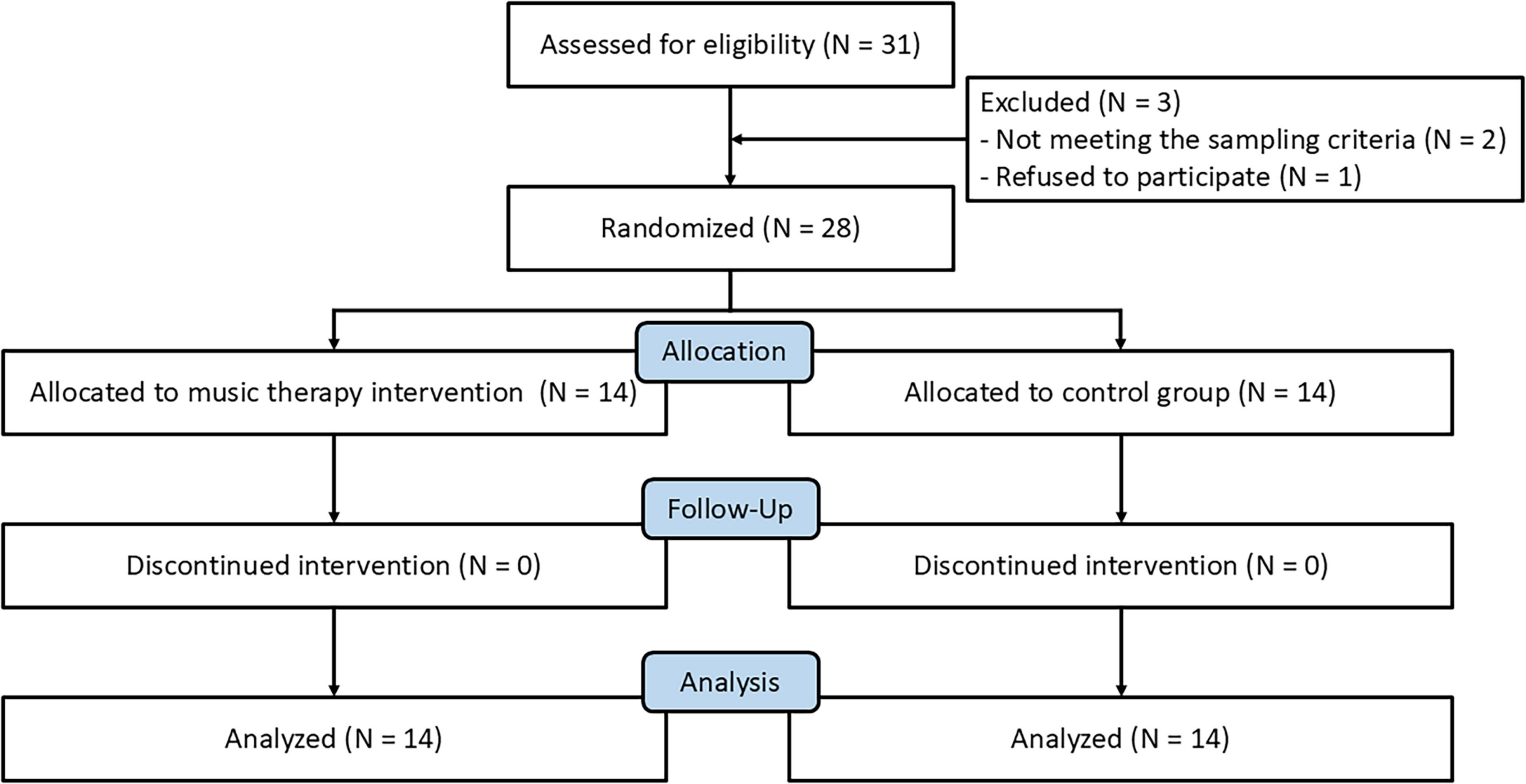
Flow diagram of the study participants.

Twenty-eight patients with schizophrenia were recruited from the community through the public health management department of a township health center in Quanzhou, Fujian Province, China (i.e., the Minnan region). Among them, patients over 30 years old were able to use Minnan dialect for daily communication, while only two younger patients were not fluent in speaking Minnan but were able to understand it. The inclusion criteria were: (1) a diagnosis of schizophrenia according to the ICD-10 criteria; (2) a disease duration of at least 8 years with stable symptoms under regular medication; (3) age between 24 and 60 years; (4) living with at least one legal guardian; and (5) voluntary participation by both patients and guardians. Exclusion criteria included: (1) severe physical comorbidities; (2) a history of alcohol or substance dependence; and (3) inability to complete interventions. Of the initially screened patients, three were excluded: two did not meet the inclusion criteria, and one declined to participate. Termination criteria during the study were defined as: (1) relapse confirmed by psychiatrists, indicated by a ≥25% increase in the total score of the Brief Psychiatric Rating Scale (BPRS) (Gorham and Overall, 1961; Zhang, 1998); (2) the development of severe physical illnesses; or (3) withdrawal of consent by patients or guardians. No participants withdrew for any of these reasons. Participants were randomly assigned, using a random number table, to either the music intervention group (*n* = 14; mean age = 39.21 years, *SD* = 7.43) or the control group (*n* = 14; mean age = 43.43 years, *SD* = 3.55). Among the 28 patients, 12 were employed (including those self-employed) and 16 were unemployed; 4 were unmarried, 13 were married, and 11 were divorced. The participant flow is illustrated in Figure 1. A *post hoc* analysis with G*Power showed that 28 participants provided excellent power (1-β = 0.993), ensuring reliable detection of effects in the analyses.

### 2.2 Study design

This was a non-equivalent control group pretest-posttest quasi-experimental design. Participants were randomly assigned to either the music intervention group or the control group by an assisting research student who was not involved in the intervention or assessment process. Participants in the control group continued with their routine family care, which included adherence to prescribed medication and regular daily activities. In contrast, all 14 participants in the music intervention group received weekly 60-minute group music therapy sessions (eight sessions over 8 weeks) together as a single group, in addition to standard family care. After the intervention period, participants in the control group were offered four optional individual music therapy sessions as compensatory care.

Each 60-minute session was implemented by one to two qualified therapists, including a board-certified Neurologic Music Therapist (certified by the Certification Board for Music Therapists, CBMT, USA) and a Level-1 Drum Circle Facilitator (certified by Voice Movement Therapy China, VMC). In addition, three to five graduate students in music therapy assisted in the sessions by preparing materials, observing, and guiding participants as needed.

### 2.3 Music therapy intervention protocol

The intervention protocol was informed by Wheeler’s three-phase framework of music therapy in psychiatric settings (Wheeler, 1983), which organizes therapeutic approaches progressively according to patients’ clinical condition, social environment, and therapeutic goals. In the present study, only the first two phases were applied due to time and session limitations.

**Phase 1**: Supportive, Activity-Oriented Music Therapy. This phase focused on the “here and now” and primarily adopted receptive music therapy methods, such as music relaxation training and song discussion. The aim was to provide a supportive environment and to help patients stabilize emotions and promote engagement through structured musical activities.

**Phase 2**: Re-educative, Introspective, and Process-Oriented Music Therapy. Building on patients’ basic sense of reality, this phase emphasized meaningful communication. It mainly applied re-creative music therapy methods, such as simple instrument playing, singing, or learning basic musical skills under the therapist’s guidance. The purpose was to facilitate self-reflection and to practice interpersonal skills through music interaction.

Each 60-minute session consisted of three segments: (1) introductory session (10–15 min), aimed at building rapport and clarifying the goals of the session; (2) active therapeutic session (35–40 min), during which different music therapy activities were implemented according to the intervention phase and time arrangement, including musical improvisation, rhythmic exercises, and collaborative music-making; and (3) closing session (10–15 min), focusing on emotional reflection and summarizing the session debriefing.

A standardized set of instruments, including piano, drums, guitar, tambourine, maracas, and triangle, was used to support the interventions. Specific activities and objectives for each therapy phase are detailed in Table 1.

**TABLE 1:** The comprehensive group music therapy intervention protocol.

Phase	Sessions	Objectives	Activities and methods
Pre-intervention		Assessment: evaluate baseline functioning (daily living skills, quality of life, emotional and cognitive status). Communication: collect medical and psychosocial history via interviews.	- Administer standardized questionnaires. - Obtain informed consent. - Conduct semi-structured interviews with patients, caregivers, and clinicians.
Phase 1 (supportive, activity-oriented)	1–3	Cognitive: enhance attention, rhythm perception, and body awareness. Emotional: facilitate emotional expression. Behavioral: reduce social isolation and foster group cohesion.	1. Hello song (group singing - recreative MT). 2. Themed song recreation (lyric/style adaptation - recreative MT). 3. Rhythmic improvisation (drum circle - improvisational MT). 4. Guided movement to music (body percussion/dance - receptive MT). 5. Goodbye song (closure ritual - recreative MT).
Phase 2 (re-educative, process-oriented)	4–6	Cognitive: strengthen reality orientation and “here-and-now” awareness. Emotional: promote emotional identification, catharsis, and positivity. Behavioral: encourage self-expression and social interaction.	1. Hello song (ritual reinforcement - recreative MT). 2. Music-assisted life review (song discussion - receptive MT). 3. Structured ensemble playing (thematic improvisation - improvisational MT). 4. Lyric analysis and rewriting (metaphor exploration - recreative MT). 5. Goodbye song (group reflection - receptive MT).
	7–8	Cognitive: deepen self/environmental awareness. Emotional: improve emotional regulation and interpersonal bonding. Behavioral: reinforce adaptive behaviors.	1. Hello song (group synchronization - recreative MT). 2. Free improvisation (emotional projection - improvisational MT). 3. Collaborative songwriting (theme: resilience - recreative MT). 4. Music-guided imagery (relaxation - receptive MT). 5. Goodbye song (affirmation sharing - recreative MT).
Post-intervention		Assessment: re-evaluate functional and emotional outcomes. Feedback: gather qualitative insights on intervention efficacy.	- Repeat baseline questionnaires. - Conduct debriefing interviews with participants and caregivers.

MT, music therapy. Each intervention session (sessions 1–8) included four core songs: Mian Shi Zhi (“Never Lose Heart”), Shi Jie Di Yi Deng (“Number One in the World”), Huan Xi Jiu Hao (“Just Be Happy”), and one song selected by participants during the request segment. In addition, the opening song (Ni Hao Ge, “Hello Song”) and the closing song (Zai Jian Ge, “Goodbye Song”) were consistently used in every session to provide a stable musical signal and establish a clear sense of ritual for the beginning and end of therapy. For detailed information on the music/songs used in each session, see Supplementary Table 1.

During the music therapy intervention process, the following points should be considered: To ensure patient safety and prevent accidents or adverse reactions, a doctor will be present at each session. Criticism and blame should be avoided; instead, patients should be encouraged to persist, and the treatment plan was designed in advance with consistent therapeutic themes, activity structure, and main objectives across the eight-week intervention period. The intervention design takes cultural background into account by incorporating classic Minnan dialect (i.e., Southern Fujian dialect) music and songs, such as Ai-Piah Cia Eh Yia (“No pain, No Gain”), Huan Xi Jiu Hao (“Just Be Happy”), and Shi Jie Di Yi Deng (“Number One in the World”). These widely known and frequently performed works are deeply embedded in the daily life and cultural identity of local residents. Selecting such songs ensured that the musical materials were familiar and readily acceptable to the participants, thereby maximizing the positive influence of cultural context. In addition, Minnan dialect-speaking teaching assistants were provided to further support communication and engagement. During the intervention, therapists monitored and noted participants’ responses to musical activities as part of the process.

### 2.4 Outcome measures

#### 2.4.1 Positive and negative affect schedule (PANAS) – primary outcome

The PANAS (Huang et al., 2003; Watson et al., 1988) was used as the primary outcome measure to assess affective changes before and after the intervention. The PANAS contains 20 items rated on a 5-point scale (1 = *very slightly or not at all* to 5 = *extremely*), yielding two subscales: Positive Affect (PA), reflecting energy and enthusiasm, and Negative Affect (NA), indicating distress and unpleasant engagement. Cronbach’s α in this study was 0.83.

#### 2.4.2 Social disability screening schedule (SDSS) – secondary outcome

The SDSS (WHO, 1988; Zhang, 1998) served as the secondary outcome measure, assessing participants’ social functioning. The SDSS includes a 10-item scale rated on a 3-point scale (0 = *no impairment*, 1 = *mild impairment*, 2 = *severe impairment*), yielding total scores ranging from 0 to 20. There are 10 items in total, including occupational role, marital function, parental function, social withdrawal, social activities, participation in household activities, family functioning, self-care, responsibility or plan and interest and attention in the environment. Cronbach’s α in this study was 0.87.

#### 2.4.3 Semi-structured interviews – supplementary qualitative assessment

A self-developed semi-structured interview was conducted as a supplementary qualitative measure to qualitatively assess the perceived impact of the music therapy intervention. The interview included 25 core questions directed at both patients and their caregivers, focusing on three key domains: (1) symptomatic experience; (2) emotional and behavioral changes; (3) social functioning and interpersonal engagement (see Supplementary Table 2). The interview aimed to capture participants’ subjective experiences and provide complementary insights to the quantitative measures.

### 2.5 Data analysis

Quantitative data were analyzed using SPSS 26.0. Continuous variables are presented as mean ± standard deviation (*M* ± *SD*), and categorical variables as frequencies. Depending on the normality of the data, either independent sample *t*-tests or Mann-Whitney *U* tests were used to compare differences in continuous variables between the two groups, while Chi-square tests were applied to examine differences in categorical variables. A series of mixed-design analysis of variance (ANOVAs) was conducted to examine changes over time and between groups for the SDSS and PANAS scales and their respective subscales, with time (pre-test, post-test) as a within-subjects factor and group (music intervention, control) as a between-subjects factor. For variables showing significant time × group interaction effects, simple effects analyses were conducted. In addition to *p*-values, effect sizes (e.g., Cohen’s *d*, η_*p*_^2^) and 95% confidence intervals were reported. A *p*-value of <0.05 was considered statistically significant.

## 3 Results

### 3.1 Demographic comparisons

Baseline comparisons between music intervention and control groups revealed no significant differences across demographic and clinical variables (*p*s. > 0.05; see Table 2). These results indicate that the two groups were comparable at baseline.

**TABLE 2:** Demographic characteristics between the music intervention and control groups.

Variable	Music intervention group (*n* = 14)	Control group (*n* = 14)	Statistic	*p*	Effect size [95% CI]
Gender (M/F)	8/6	7/7	χ^2^ (1) = 0.14	0.705	–
Education level (≤ junior high/ ≥ high school)	11/3	11/3	χ^2^ (1) = 0.00	1.000	–
Age (years)	39.21 ± 7.43	43.43 ± 3.55	*t* (26) = −1.92	0.067	−0.72 [−1.48, 0.05]
Duration of illness (years)	16.93 ± 7.64	16.00 ± 6.35	*U* = 90.00	0.729	−0.08 [−0.48,0.34]
BPRS	29.36 ± 8.10	32.21 ± 13.48	*U* = 92.00	0.800	−0.06 [−0.46,0.36]

M, male; F, female. For the student *t*-test, effect size is reported as Cohen’s *d*, and for the Mann-Whitney *U* test, effect size is reported as the rank biserial correlation.

### 3.2 Comparison of emotional states between groups across interventions

**FIGURE 2:**
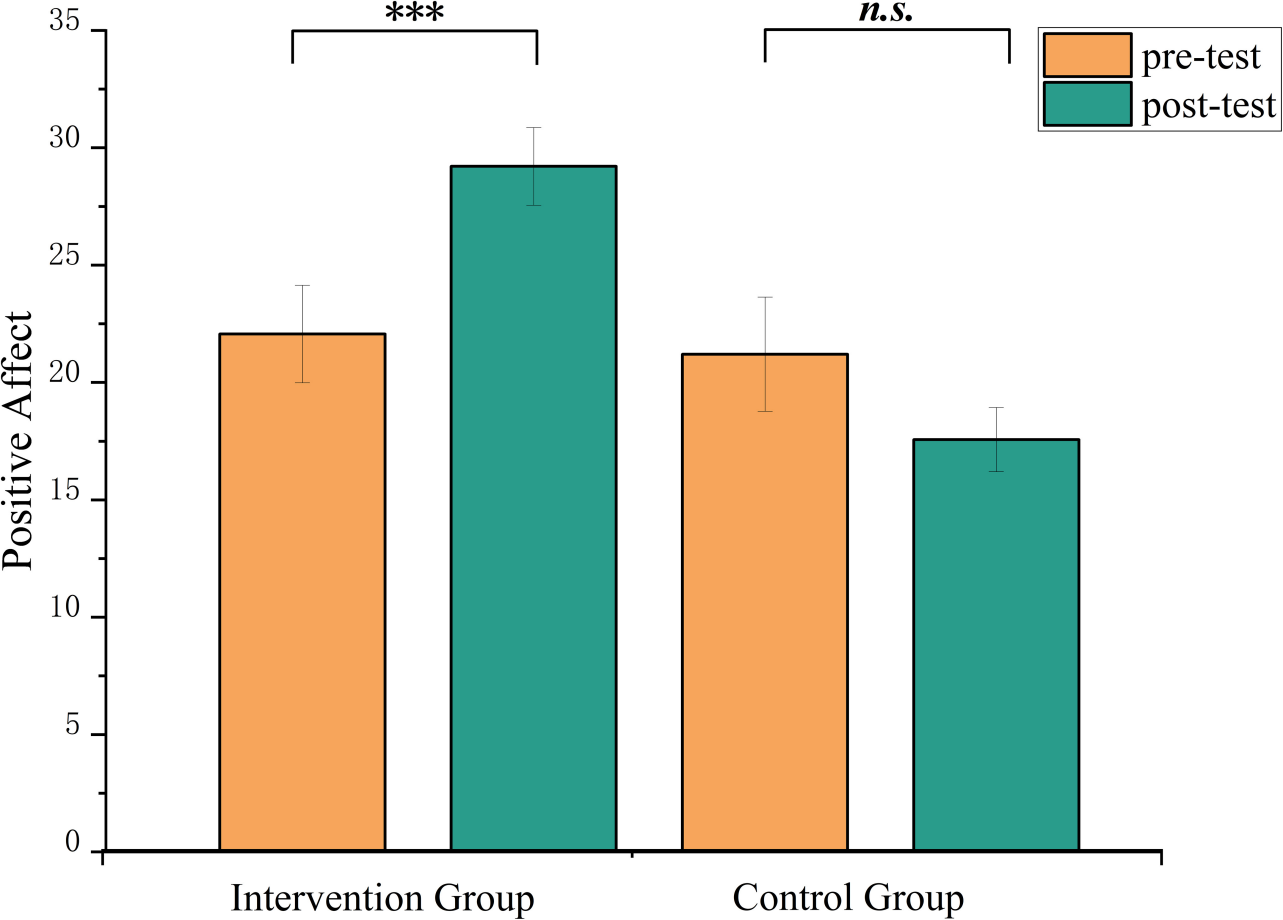
Changes in positive emotion of PANAS scores in the music intervention and control group before and after the intervention. ***p < 0.001. n.s., non-significant.

A series of mixed-design ANOVAs was conducted with group (music intervention vs. control) × time (pre-test vs. post-test) as independent variables, using scores of positive affect and negative affect from the PANAS as dependent variables. As presented in Table 3, a significant group × time interaction was observed for positive affect, *F*(1, 26) = 7.19, *p* = 0.013, η_*p*_^2^ = 0.22. Simple effect analysis indicated no significant difference between groups at pre-test (22.07 ± 7.76 vs. 21.21 ± 9.11), *t* = 0.32, *p*bonf = 1.000. However, at post-test, the music intervention group exhibited significantly higher positive affect scores (29.21 ± 6.20) compared to the control group (17.57 ± 5.11), *t* = 4.27, *p*bonf <0.001 (see Figure 2). To further verify the robustness of these findings and to avoid the inflation of Type I error due to multiple comparisons, a MANOVA was performed using the difference scores of positive and negative affect as dependent variables. The multivariate test remained significant, Wilks’ λ = 0.72, *F*(2, 25) = 4.98, *p* = 0.015, η_*p*_^2^ = 0.29.

**TABLE 3:** Changes in PANAS scores in the music intervention and control group before and after the intervention.

Variables	Main effect of time			Main effect of group			Interaction effect		
	*F*(1, 26)	*p*	η_p_^2^	*F*(1, 26)	*p*	η_p_^2^	*F*(1, 26)	*p*	η_p_^2^
Positive affect	0.76	0.392	0.03	11.57	0.002	0.31	**7.19**	**0.013**	**0.22**
Negative affect	4.74	0.039	0.15	3.80	0.062	0.13	0.66	0.424	0.03

Significant interactions between group and time (*p* < 0.05) are marked in bold.

### 3.3 Comparison of social functioning between groups

A series of mixed-design ANOVAs was conducted with group (music intervention vs. control) × measurement time (pre-test vs. post-test) as independent variables, and the total score and subscale scores of the SDSS as dependent variables. As shown in Table 4, a significant interaction effect was observed for the social activities subscale [*F*(1, 26) = 5.06, *p* = 0.033, η_*p*_^2^ = 0.16] and the self-care subscale [*F*(1, 26) = 5.44, *p* = 0.028, η_*p*_^2^ = 0.17], suggesting differential changes over time between the two groups on these variables.

**TABLE 4:** Changes in SDSS scores in the music intervention and control group before and after the intervention.

Variables	Main effect of time			Main effect of group			Interaction effect		
	*F*(1,26)	*p*	η_p_^2^	*F*(1,26)	*p*	η_p_^2^	*F*(1,26)	*p*	η_p_^2^
SDSS	3.78	0.063	0.13	5.49	0.027	0.17	2.46	0.129	0.09
Occupational role	2.95	0.098	0.10	3.24	0.083	0.11	0.86	0.364	0.03
Marital function	0.51	0.483	0.02	0.74	0.398	0.03	0.13	0.725	0.01
Parental function	1.38	0.250	0.05	2.37	0.136	0.08	0.25	0.619	0.01
Social withdrawal	3.01	0.095	0.10	4.73	0.039	0.15	3.01	0.095	0.10
Social activities	5.06	0.033	0.16	8.21	0.008	0.24	**5.06**	**0.033**	**0.16**
Participation in household activities	1.28	0.268	0.05	1.11	0.302	0.04	0.57	0.458	0.02
Family functioning	0.38	0.541	0.02	3.13	0.089	0.11	3.45	0.075	0.12
Self-care	0.05	0.834	0.00	0.38	0.541	0.02	**5.44**	**0.028**	**0.17**
Interest and attention in the environment	1.28	0.268	0.05	0.16	0.695	0.01	0.72	0.404	0.03
Responsibility or plan	6.13	0.020	0.19	1.05	0.315	0.04	0.53	0.473	0.02

Significant interactions between group and time (*p* < 0.05) are marked in bold.

**FIGURE 3:**
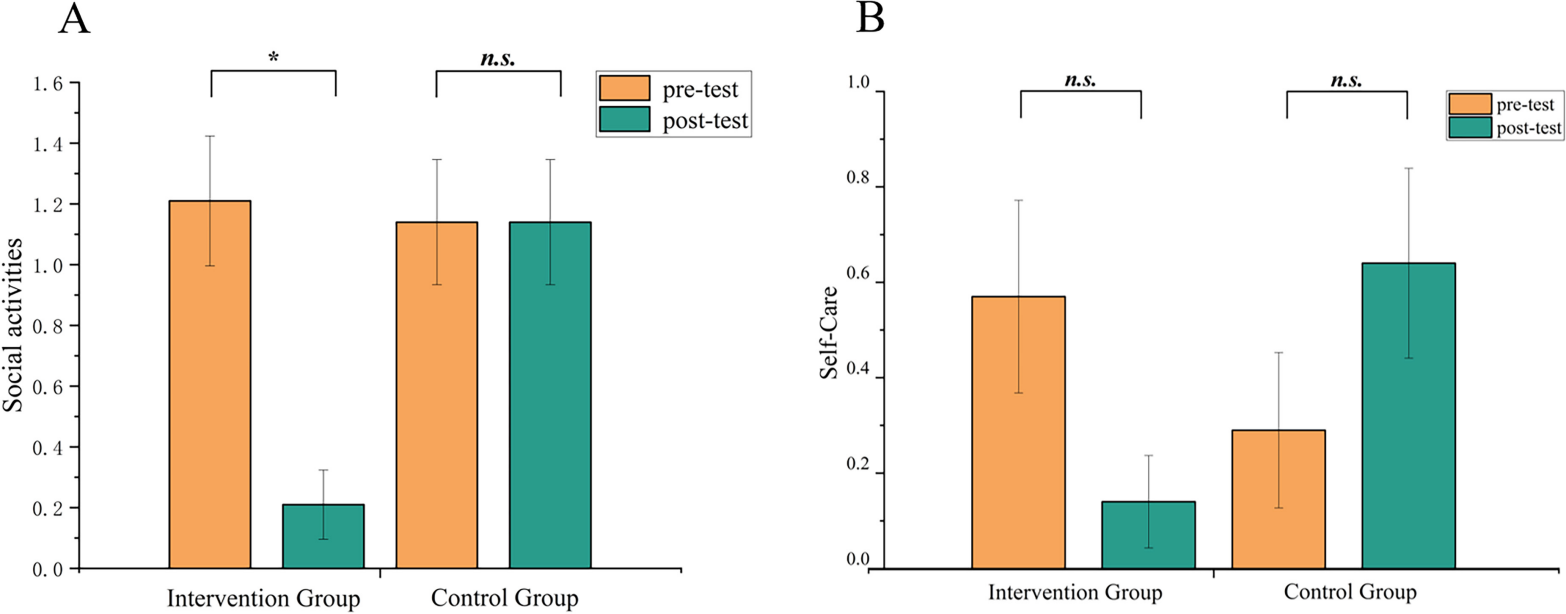
Changes in the social activities **(A)** self-care **(B)** scores of SDSS in the music intervention and control group before and after the intervention. *p < 0.05. n.s., non-significant.

For social activities, simple effect analysis revealed that the intervention group showed a significant reduction from pre-test (1.21 ± 0.80) to the post-test (0.21 ± 0.43), *t* = 3.18, *p*bonf = 0.023, while the control group did not exhibit any significant difference between pre-test (1.14 ± 0.77) and post-test (1.14 ± 0.77), *t* = −1.765 × 10^15^, *p*bonf = 1.000. No significant difference was found between the pre-test scores of the intervention group (1.21 ± 0.80) and the control group (1.14 ± 0.77), *t* = 0.27, *p*bonf = 1.000, but the post-test score of the intervention group (0.21 ± 0.43) was significantly lower than that of the control group (1.14 ± 0.77), *t* = −3.47, *p*bonf = 0.007 (see Figure 3A).

For self-care, the intervention group’s pre-test score (0.57 ± 0.76) was higher than the post-test score (0.14 ± 0.36), while the control group’s pre-test score (0.29 ± 0.61) was lower than the post-test score (0.64 ± 0.75). However, simple effect analysis did not reveal any significant differences in either group (*p*s. > 0.05) (see Figure 3B).

Additionally, a significant main effect of group was found for the SDSS total score, *F*(1, 26) = 5.49, *p* = 0.027, η_p_^2^ = 0.17, with the intervention group (5.82 ± 5.05) being significantly lower than the control group (8.14 ± 4.56). For the social withdrawal subscale, a significant main effect of group was observed, *F*(1, 26) = 4.73, *p* = 0.039, η_p_^2^ = 0.15, with the intervention group (0.71 ± 0.68) being significantly lower than the control group (1.14 ± 0.82). For the responsibility or plan subscale, a significant main effect of time was found, *F*(1, 26) = 6.31, *p* = 0.020, η_p_^2^ = 0.19, with the post-test score (0.68 ± 0.77) being significantly lower than the pre-test score (1.29 ± 0.83).

To address the issue of multiple univariate tests, we also performed a MANOVA using the difference scores (post − pre) of the SDSS subscales as dependent variables and group as the independent variable. The multivariate test did not reach significance, Wilks’ λ = 0.72, *F*(10, 17) = 1.26, *p* = 0.323, η_p_^2^ = 0.43. Therefore, the above univariate subscale findings should be interpreted with caution.

### 3.4 Follow-up interviews

One week after the intervention, follow-up group interviews were conducted with a subset of eight participants from the music intervention group and their family members (see Supplementary Table 3).

Overall, all eight participants reported significant improvements in mood, describing feelings of relaxation and enjoyment during activities such as singing and dancing. Seven participants were able to clearly recall details from the sessions and reported forming positive associations with the experience. Specifically, certain activities, such as the drum circle performances and singing, were especially enjoyed by the participants, with one stating, “*I feel much better after participating.*” Family members also observed important changes, noting that the patients, who had previously yelled in the yard to release emotions, now displayed more stable emotional states and rarely used shouting as an emotional outlet. Positive emotional levels were significantly improved. One patient shared, “*At home, I also listen to music and move to the rhythm. Before sleeping, I even drum on my belly to create a rhythm.*”

In terms of social functioning, seven patients became more socially engaged with their family members after the activities. Family members confirmed the positive behavioral changes, observing that patients transitioned from being silent to actively sharing the details of the activities. One patient, in particular, exhibited a noticeable shift, as they proactively bought yogurt for their child after returning home - an action that family members had never seen before. The intervention also effectively enhanced motivation for social participation, with all participants expressing a desire to continue attending the sessions. Three participants even voluntarily recommended the program to their neighbors.

However, it is important to highlight the limitations in addressing psychiatric symptoms. While one patient described, “*When I participated in the music therapy, the voices disappeared*,” it’s important to note that this patient had very severe auditory hallucinations, which were a source of significant distress. Although their hallucinations did not appear during the therapy sessions and symptoms were alleviated at that time, the overall improvement in psychiatric symptoms was limited.

## 4 Discussion

This study investigated the effects of a culturally adapted comprehensive group music therapy program on affect and social functioning in individuals with schizophrenia undergoing community-based rehabilitation. The results indicated that, compared to individuals in the control group, those receiving music therapy exhibited a significant increase in Positive Affect as assessed by the PANAS, and a significant improvement in social activities and self-care as measured by the SDSS subscale. These findings suggest that group therapy may be a promising adjunctive intervention for enhancing both emotional state and social functioning in this population.

Regarding emotional outcomes, this study corroborates existing evidence that music therapy can facilitate emotional regulation, alleviate tension, and promote the expression of positive emotions across various clinical populations (James et al., 2020), including individuals with schizophrenia (Lu et al., 2013). In our sample, participants receiving music therapy showed significant improvements in positive affect, and qualitative interviews further supported these quantitative findings - most participants reported subjective improvements in mood and emotional awareness following the intervention. These results suggest that group music therapy may foster emotional well-being and resilience among individuals with schizophrenia engaged in community-based rehabilitation.

In terms of social functioning, our findings are consistent with previous studies demonstrating the beneficial effects of music therapy on functional outcomes among hospitalized individuals with schizophrenia (Lee and Lee, 2020). Although no significant improvements were found in overall SDSS scores, and MANOVA on all subscale difference scores did not yield significant results, clear effects were observed in the social activities and self-care domains. Similar trends were also noted in the SDSS total score, as well as the social withdrawal and responsibility or plan subscales, where the intervention group exhibited improvement, whereas the control group did not, although these differences did not reach statistical significance. These discrepancies may partly stem from differences in assessment tools; for instance, Lee and Lee (2020) employed the Independent Living Skill Survey (Wallace et al., 2000), which specifically targets instrumental skills required for independent community living, whereas the SDSS used in this study evaluates broader social role functioning. Furthermore, improvements in social activities and self-care may be more readily observable in community-based rehabilitation settings compared to inpatient environments, where patients’ opportunities for independent social activities and self-care are inherently limited. Interview data supported these findings: patients became more socially engaged with family members and showed increased motivation for social participation. For example, one patient proactively bought yogurt for their child for the first time, and several participants expressed a desire to continue attending the sessions. These contextual differences highlight the value of tailoring both interventions and outcome assessments to specific rehabilitation stages.

In addition, cultural adaptation emerged as a critical factor influencing the effectiveness of the intervention. Prior research has highlighted the importance of cultural relevance in designing psychosocial interventions for schizophrenia (Ngubane et al., 2024). For example, Ngubane et al. (2024) demonstrated that incorporating traditional Korean music into music therapy enhanced emotional and interpersonal outcomes among hospitalized patients with schizophrenia. Similarly, in our study, the music therapy program integrated local Minnan dialect music and songs and employed Minnan-speaking assistants to create a familiar and culturally resonant therapeutic environment. More than 85% of participants and their family members reported that the cultural relevance of the intervention contributed substantially to their engagement and comfort during therapy sessions. This cultural adaptation not only enhanced participants’ emotional engagement but also created a sense of cultural resonance, which may have contributed to a stronger therapeutic alliance and improved outcomes. This finding reinforces the importance of cultural sensitivity in designing community-based mental health interventions, particularly in diverse and culturally rich settings. Furthermore, it underscores the need for cultural sensitivity in research, as adapting interventions to the participants’ cultural background can significantly enhance the effectiveness and acceptance of therapeutic interventions.

There are several limitations to this study. First, the sample was relatively small and drawn from a single community. Given the low prevalence of schizophrenia in the general population, recruitment was challenging, and the findings may not generalize to other settings or populations. Future studies should aim to replicate these results in larger and more diverse samples, including across different cultural contexts, to assess the generalizability of the findings. Second, the study design had several limitations. It included only immediate pre- and post-intervention assessments and lacked mid- or long-term follow-ups, making it difficult to determine whether the observed improvements were sustained over time. In addition, potential confounding factors such as age of onset and medication status were not controlled, and no placebo or active control condition was included, which may have influenced treatment outcomes. Accordingly, our findings should be interpreted as exploratory, and causality cannot be inferred. Future research should examine whether the effects of music therapy vary according to illness stage, medication status, or demographic characteristics, incorporate placebo or alternative control interventions, and consider longitudinal designs to assess the sustainability of observed improvements. Third, the assessment dimensions were limited. The SDSS focused mainly on self-care, and emotional assessment was restricted to positive and negative affect, which may not capture the full range of social and emotional outcomes. Future studies should include broader measures, such as interpersonal, occupational, community functioning, and specific emotions like anxiety and depression. Additionally, the findings from this study are culturally specific, as they were conducted within the Minnan region, and may not apply to other cultural settings. Further research should examine whether similar effects are observed in different cultural contexts, which would help to assess the broader applicability of the intervention.

## 5 Conclusion

In conclusion, integrating local cultural elements into music therapy may enhance emotional state and social functioning among individuals with schizophrenia in community-based rehabilitation, highlighting the importance of culturally sensitive interventions in mental health care. As the bio-psycho-social model (Engel, 1977) continues to shape contemporary approaches to mental health care, the value of community-based psychological services is increasingly recognized. Group music therapy contributes valuable insights and innovative possibilities within this framework. Moving forward, it is crucial not only to further examine the therapeutic mechanisms of specific musical components (Chen et al., 2022), but also to consider the cultural relevance and contextual fit of interventions. Culturally attuned music therapy can foster greater patient engagement, promote a sense of belonging, and ultimately enhance intervention outcomes and quality of life for individuals with schizophrenia living in community settings.

## Data Availability

The raw data supporting the conclusions of this article will be made available by the authors, without undue reservation.
