# Supplementary material for: Effects of comprehensive group music therapy on affect and social functioning in patients with schizophrenia undergoing community-based rehabilitation: a preliminary study
Source: Front Hum Neurosci. 2025 Nov 26;19:1645981. doi: 10.3389/fnhum.2025.1645981 (PMC12689967; doi:10.3389/fnhum.2025.1645981)
Supplement: Supplementary file 1 [file Table_1.docx]

Supplementary Material

# Supplementary Tables

**Supplementary Table 1.** Music intervention sessions and activities.

| session | Activity | Duration |
| --- | --- | --- |
| 1 | 1. “Hello Song”(你好歌)  2. Dance “Never Lose Heart” (《免失志》)  3. Singing “Number One in the World”(《世界第一等》)  4. Song Request  5. Dance “Just Be Happy” (《欢喜就好》)  6. “Goodbye Song”(再见歌) | 7 min  10 min  10 min  15 min  10 min  8 min |
| 2 | 1. “Hello Song”(你好歌)  2. Dance “Never Lose Heart” (《免失志》)  3. Singing “Number One in the World”(《世界第一等》)  4. Song Request  5. Dance “Just Be Happy” (《欢喜就好》)  6. “Goodbye Song” (再见歌) | 7 min  10 min  10 min  15 min  10 min  8 min |
| 3 | 1. “Hello Song”(你好歌)  2. Dance “Never Lose Heart” (《免失志》)  3. Singing & Improvised Instrumental “Above the Moon”(《月亮之上》)  4. Song Request  5. Drum Circle Improvisation  6. Dance “Just Be Happy” (《欢喜就好》)  7. “Goodbye Song” (再见歌) | 6 min  8 min  12 min  10 min  15 min  6 min  3 min |
| 4 | 1. “Hello Song”(你好歌)  2. Dance “Just Be Happy” (《欢喜就好》)  3. Movement Therapy  Warm-up Exercise “Basamemucho”  Body Sensory Training “paris in the rain”  Dynamic Balance “Empty love”  Body Control Training “Ninna Nanna”  Integration Review “Magic Potion” (《给你一瓶魔法药水》)  4. “Goodbye Song” (再见歌) | 5 min  8 min  40 min  7 min |
| 5 | 1. “Hello Song”(你好歌)  2. Dance “Never Lose Heart” (《免失志》)  3. Movement Therapy  Warm-up Exercise “Basamemucho”  Body Sensory Training “paris in the rain”  Dynamic Balance “Empty love”  Body Control Training “Ninna Nanna”  Integration Review “Magic Potion” (《给你一瓶魔法药水》)  4. Song Request  5. Sing “No pain, No Gain” (《爱拼才会赢》)  6. Drum Circle Improvisation  7. Dance “Just Be Happy” (《欢喜就好》)  8. “Goodbye Song” (再见歌) | 5 min  7 min  30 min  8 min  7 min  10 min  5 min  3 min |
| 6 | 1. “Hello Song”(你好歌)  2. Dance “Never Lose Heart” (《免失志》)  3. Song Lyric Writing “Peach Blossom” (《桃花朵朵开》)  4. Song Request  5. Drum Circle Improvisation  6. Singing & Improvised Instrumental “No pain, No Gain” (《爱拼才会赢》)  7. Dance “Just Be Happy” (《欢喜就好》)  8. “Goodbye Song” (再见歌) | 6 min  8 min  18 min  9 min  10 min  12 min  6 min  5 min |
| 7 | 1. “Hello Song”(你好歌)  2. Dance “Never Lose Heart” (《免失志》)  3. Song Lyric Writing “Peach Blossom” (《桃花朵朵开》)  4. Song Request  5. Drum Circle Improvisation  6. Singing & Improvised Instrumental “Descendants of the Dragon” (《龙的传人》)  7. Dance “Just Be Happy” (《欢喜就好》)  8. “Goodbye Song” (再见歌) | 6 min  8 min  18 min  9 min  10 min  12 min  6 min  5 min |
| 8 | 1. “Hello Song”(你好歌)  2. Dance “Never Lose Heart” (《免失志》)  3. Song Request  4. Drum Circle Improvisation  5. Dance “Just Be Happy” (《欢喜就好》)  6. “Goodbye Song” (再见歌) | 7 min  8 min  20 min  15 min  17 min  13 min |

**Supplementary Table 2**. Music therapy intervention effect interview guide.

| **Module** | **Interview questions** |
| --- | --- |
| 1. Symptomatic experience | 1. Have you experienced any side effects from medical treatment (e.g., dizziness, drowsiness)?  2. How many times have you been hospitalized due to illness? What were the main symptoms (such as insomnia, auditory hallucinations, aggressive behavior)?  3. Have you ever experienced physical discomfort during activities, such as dizziness? Did it affect your ability to participate?  4. (For individual with long-term auditory hallucinations) Did participation in activities help reduce the frequency or intensity of the voices? Did the symptoms return after going home?  5. (For individuals with medication side effects) Did the activities help relieve physical discomfort caused by medication, such as headaches? |
| 2. Emotional and behavioral changes | 1.What activities have you participated in (e.g., singing, dancing, and drumming)? Which part did you enjoy the most, and why?  2. What aspects of the activities made you feel uncomfortable or bored? Please give examples (e.g., repetitive songs, difficult movements).  3. Can you remember any specific songs or dance moves? Do these remind you of any personal life experiences?  4. After participating in the activities, did you notice any emotional changes (e.g., feeling happier or more depressed)? How long did these changes last?  5. Did you feel distracted during the activities? What factors contributed to your distraction?  6. What new elements would you like to see added to the activities (e.g., musical improvisation, adaptations of pop music)?  7. If you were to rate the activity from 0 to 10, what score would you give? What are the reasons for any point deductions? |
| 3. Social functioning and interpersonal engagement | 1. What is your family’s main source of income? Are you currently facing any financial stress?   2. Have you shared your experience in these activities with family or friends? How did they respond?  3. Are your family members aware of your condition? How do they support you (e.g., reminding you to take medication, accompanying you to appointments)?  4. Do you have any conflicts with family members (e.g., issues between in-laws, financial disagreements)? How do you usually handle them?  5. How are your daily interactions with others (e.g., neighbors, colleagues)? Have you ever felt discriminated against due to your illness?  6. If these activities were held regularly, would you be willing to participate? What factors might prevent you from joining (e.g., transportation, scheduling)?  7. Would you recommend this activity to other patients? Why or why not?  8. (For those with family conflicts) Do you think these activities helped improve communication with your family? |

**Supplementary Table 3.** Interview summary on the effects of music therapy intervention.

| **Module** | **Interview questions** | **Yes/No/No answer** | **Remarks** |
| --- | --- | --- | --- |
| 1. Symptomatic experience | 1. Have you experienced any side effects from medical treatment (e.g., dizziness, drowsiness)? | 7/1/0 | Seven patients reported side effects such as dizziness, headache, and repetitive thinking. |
|  | 2. How many times have you been hospitalized due to illness? What were the main symptoms (such as insomnia, auditory hallucinations, aggressive behavior)? | 6/0/2 | All hospitalized due to insomnia, auditory hallucinations, and aggressive behavior. |
|  | **3. Have you ever experienced physical discomfort during activities, such as dizziness? Did it affect your ability to participate?** | 2/5/1 | No one felt unwell during the activity, but symptoms recurred after returning home. |
|  | **4. (For individual with long-term auditory hallucinations) Did participation in activities help reduce the frequency or intensity of the voices? Did the symptoms return after going home?** | 3/3/2 | Hallucinations were alleviated during activity but relapsed after returning home. |
|  | 5. (For individuals with medication side effects) Did the activities help relieve physical discomfort caused by medication, such as headaches? | 3/2/3 | Three patients reported relief from headache or back pain. |
| 2. Emotional and behavioral changes | **1.What activities have you participated in (e.g., singing, dancing, and drumming)? Which part did you enjoy the most, and why?** | 8/0/0 | All participants enjoyed activities, felt “happy” and “relaxed.” |
|  | **2. What aspects of the activities made you feel uncomfortable or bored? Please give examples (e.g., repetitive songs, difficult movements).** | 3/4/1 | Three found songs repetitive, like “Chinese People” and “Peach Blossoms in Bloom”. |
|  | **3. Can you remember any specific songs or dance moves? Do these remind you of any personal life experiences?** | 7/0/1 | Most recalled songs (e.g., Avoid Losing Heart) and reported positive associations. |
|  | **4. After participating in the activities, did you notice any emotional changes (e.g., feeling happier or more depressed)? How long did these changes last?** | 8/0/0 | All felt emotionally improved but reported the effect was short-term. |
|  | **5. Did you feel distracted during the activities? What factors contributed to your distraction?** | 5/2/1 | Two were distracted by others’ behavior or the environment. |
|  | **6. What new elements would you like to see added to the activities (e.g., musical improvisation, adaptations of pop music)?** | 2/6/0 | Two suggested adding pop songs or new dances. |
|  | **7. If you were to rate the activity from 0 to 10, what score would you give? What are the reasons for any point deductions?** | 5/1/2 | Five scored 7–10; one gave 3 due to repetition. |
| 3. Social functioning and interpersonal engagement | 1. What is your family’s main source of income? Are you currently facing any financial stress? | 5/0/3 | Five expressed anxiety due to low income and living subsidies. |
|  | **2. Have you shared your experience in these activities with family or friends? How did they respond?** | 2/6/0 | Six never shared; two shared with close relatives. |
|  | 3. Are your family members aware of your condition? How do they support you (e.g., reminding you to take medication, accompanying you to appointments)? | 7/0/1 | Most families supported by reminding about meds or hospital visits. |
|  | 4. Do you have any conflicts with family members (e.g., issues between in-laws, financial disagreements)? How do you usually handle them? | 6/2/0 | Two had conflicts (mother-in-law/daughter-in-law, father/son, finances). |
|  | 5. How are your daily interactions with others (e.g., neighbors, colleagues)? Have you ever felt discriminated against due to your illness? | 2/4/2 | Four avoided social interaction due to hallucinations or illness stigma. |
|  | **6. If these activities were held regularly, would you be willing to participate? What factors might prevent you from joining (e.g., transportation, scheduling)?** | 8/0/0 | All were willing to continue participation. |
|  | **7.** Would you recommend this activity to other patients? Why or why not? | 3/3/2 | Three were reluctant due to fear of aggression or boredom. |
|  | **8. (For those with family conflicts) Do you think these activities helped improve communication with your family?** | 7/1/0 | One patient said there was no improvement. |

*Note*: The questions related to music therapy are marked in bold.
